# Exosomal LncRNA RP5-977B1 as a novel minimally invasive biomarker for diagnosis and prognosis in non-small cell lung cancer

**DOI:** 10.1007/s10147-022-02129-5

**Published:** 2022-04-28

**Authors:** Ling Min, Ting Zhu, Bo Lv, Taixue An, Qichao Zhang, Yanyan Shang, Zhiwu Yu, Lei Zheng, Qian Wang

**Affiliations:** 1 Laboratory Medicine Center, Nanfang Hospital, Southern Medical University, No. 1838 Guangzhouda Road, Guangzhou, 510515 Guangdong China; 2 Department of Laboratory Medicine, Affiliated Cancer Hospital and Institute of Guangzhou Medical University, Guangzhou, 510095 Guangdong China; 3 Department of General Practice, Guangdong Provincial Geriatrics Institute, Guangdong Provincial People’s Hospital, Guangdong Academy of Medical Sciences, Guangzhou, 510080 Guangdong China; 4 The Second School of Clinical Medicine, Southern Medical University, Guangzhou, 510080 Guangdong China

**Keywords:** RP5-977B1, NSCLC, Exosomes, Diagnosis, Prognosis

## Abstract

**Background:**

Lung cancer is the leading cause of cancer-related deaths in the world. Non-small cell lung cancer (NSCLC) accounts for 85% of all lung cancer cases. For lack of conveniently sensitive and specific biomarkers, the majority of patients are in the late stage at initial diagnosis. Long non-coding RNAs (LncRNAs), a novel type of non-coding RNA, have recently been recognized as critical factors in tumor initiation and progression, but the role of exosomal LncRNAs has not been thoroughly excavated in NSCLC yet.

**Methods:**

We isolated exosomes from the serum of patients with NSCLC and healthy controls. Exosome RNA deep sequencing was subsequently performed to detect differentially expressed exosomal LncRNAs. qRT-PCR assay was then utilized to validate dysregulated LncRNAs in both testing and multicentric validation cohort. Receiver operating characteristic (ROC) curve was used to detect the diagnostic capability of exosomal biomarkers. Furthermore, Kaplan–Meier analysis was applied to evaluate the prognostic values of these molecules.

**Results:**

On the basis of analysis, we found that novel exosomal LncRNA RP5-977B1 exhibited higher levels in NSCLC than that in the healthy controls. The area under the curve (AUC) value of exosomal RP5-977B1 was 0.8899 and superior to conventional biomarkers CEA and CYFRA21-1 both in testing and multicentric validation cohort. Interestingly, the diagnostic capability of exosomal RP5-977B1 was also validated in early-stage patients with NSCLC. Furthermore, high expression of exosomal RP5-977B1was closely related with worse prognosis in NSCLC (*P* = 0.036).

**Conclusions:**

Our results suggested that exosomal RP5-977B1 might serve as a novel “liquid biopsy” diagnostic and prognostic biomarker to monitor NSCLC and improve possible therapy.

**Supplementary Information:**

The online version contains supplementary material available at 10.1007/s10147-022-02129-5.

## Introduction

Lung cancer (LC) is a leading cause of cancer-related mortality worldwide. Among all lung cancer cases, more than 80% are non-small cell lung cancer (NSCLC), which can be further subtyped into lung adenocarcinoma (LAD), lung squamous cell carcinoma (LSCC), large cell carcinoma (LCC), and other relatively less frequently diagnosed histological types[1, 2]. Being diagnosed at an advanced stage with a high recurrence rate, the overall 5-year survival rate is below 15%, and the prognosis for the majority of patients is far from satisfactory [3]. Thus, it is urgent to develop novel and effective markers for early diagnosis and prognosis prediction.

Exosomes are secreted membranous vesicles with a size of 30–150 nm [4, 5]. With inward budding of late endosomes, exosomes evolve into internal multivesicular endosomes (MVEs) [4, 6]. In this process, bioactive factors such as DNAs, RNAs, and proteins are encapsulated into exosomes[7]. Notably, RNAs are reported to be the main components of tumor cell-derived exosomes, which can reflect tumor progression and dynamic process of tumor cells [5, 8]. Furthermore, when released into the extracellular environment and enter the peripheral blood system, exosomal RNAs are protected from degradation by endogenous RNases and, thus, increased the stability in the blood stream [9]. Therefore, circulating exosomal RNAs are emerging as the promising biomarkers for early monitoring of cancer and prognostic evaluation of patients.

Long non-coding RNAs (LncRNAs) are a heterogeneous class of transcripts longer than 200 nucleotides in size without coding potential [10–14]. It is found that LncRNAs are abundant in whole blood and involved in carcinogenesis and development of many malignant tumors, including NSCLC [15–17]. In the present study, we focused on exosomal LncRNAs to explore its value for early diagnosis and prognostic assessment in NSCLC.

## Materials and methods

### Patients and clinical samples

As a testing cohort, a total of 178 cases of NSCLC and the healthy serum samples between January 2016 and August 2019 are enrolled from Cancer Center of Guangzhou Medical University (CCGMU). 156 cases of validation cohort was comprised of patients with early-stage NSCLC and recruited from Sun Yat-Sen University Cancer Center, Nanfang Hospital, Southern Medical University and the First Affiliated Hospital of Guangzhou Medical University between April 2017 and January 2020. Benign lung diseases including pulmonary tuberculosis was recruited from Guangzhou Chest Hospital between August and October 2019.

Patients who accepted chemotherapy or radiotherapy before collection were excluded. For the use of these clinical materials for research purposes, prior patient consents and approval from the Institutional Research Ethics Committee of Southern Medical University were obtained. The research was carried out according to the principles set out in the Declaration of Helsinki 1964 and all subsequent revisions, informed consent was obtained, and the relevant institutional review board had approved the study. Clinical information of the samples is described in detail in Table 5.

### Exosomal RNA sequencing

Exosomal RNA sequencing was detected by RiboBio biotech company (Guangzhou, China). In briefly, Exosomal RNA was retrotranscribed and amplified to double stranded cDNA and followed by adaptor ligation and enrichment as according to standard protocol of NEBNext^®^ Ultra™ Directional RNA Library Prep Kit. The library products were validated by Agilent 2200 Tape Station (Agilent Technologies) and a Qubit^®^ 2.0 Fluorometer (Life Technologies) and then diluted on a HiSeq3000 paired-end flow cell followed by sequencing (HiSeq3000 system, 2 × 150 bp).

### Exosomes isolation and characterization

Exosomes were isolated from human serum as previously described [18]. Briefly, 5 ml of serum was thawed on ice, diluted in 1 × phosphate-buffered saline (PBS) (1:10), and pre-cleared using a 0.22 μm pore filter. Then, the samples were ultracentrifuged at 150,000*g* overnight at 4 °C. The supernatant solution was discarded, the remaining pellet was washed in 11 ml 1 × PBS followed by ultracentrifugation at 150,000*g* at 4 °C for 2 h. The exosomes pellet was resuspended in Trizol for RNA isolation, in lysis buffer for WB, or in PBS for transmission electron microscopy (JEM-1200EX, Japan) and Flow NanoAnalyzer (NanoFCM Inc, China). For qRT-PCR assay, exosomes were isolated and purified according to the manufacturer’s instructions using exoEasy Maxi Kit (Qiagen, Germany).

### Nanoparticle tracking analysis

Nanoparticle tracking analysis (NTA) was assessed as previously described using the Flow NanoAnalyzer (NanoFCM Inc). After the ultracentrifuge, all the samples were monitored with the use of same injection pressure (1.5 kPa) for 60 s. The samples were diluted at appropriate multiples results in approximately 1000–6000 particles per minute. The process was repeated three times. NTA software was used to measure the size and the concentration of nanoparticles.

### Western blotting analysis

Western blotting analysis was performed according to a previously described standard method using the antibodies anti-CD63 (1:1000, Abcam, MA) and anti-CD 9 (1:1000, Abcam) [19]. The blotted membranes were stripped and re-blotted with anti-α-tubulin (1:3000, Abcam) as a loading control.

### RNA extraction from exosomes

Total exosomal RNA was isolated using HiPure Exosome RNA Kit (Qiagen) following the standard protocol provided by the manufacturer. RNA purity and concentration was quantified using a Nanodrop^®^ ND-1000 (Thermo Fischer Scientific, MA).

### Total RNA isolation

Total RNA was extracted by TRIzol LS Reagent (Invitrogen, Carlsbad, CA) as previously described [19]. Briefly, 500 μl of serum was mixed with an equal volume of TRIzol LS Reagent, incubated for 5 min on ice. Subsequently, 1000 μl of Acid-Phenol: Chloroform (Invitrogen) was added, vortexed and centrifuged for 25 min at 20,000*g*. The aqueous (upper) phase was collected, and 1.25 volumes of 100% ethanol were added. The RNA was then purified with miRNeasy Mini Kit (Qiagen) in accordance with the manufacturer’s instructions. The RNA concentration was assessed using a NanoDrop ND-1000 instrument (Thermo Fisher Scientific).

### Quantitative real-time-PCR (qRT-PCR)

First-strand cDNA was generated from 500 ng of serum circulating RNA using MMLV transcriptase (Promega, WI). qRT-PCR was performed on a CFX96 qRT-PCR detection system (Bio-Rad, Richmond, CA). The expression levels were measured using the $2^{ - \Delta \Delta C_{{\text{t}}}}$ ($C_{{\text{t}}}$ is threshold cycle) formula. The sequences of the primers are listed below:

- RP5-977B1: 5′-TTTGAGGATGCGGGTGAA-3′ (forward)
- 5′-ATGAGGAAGTGGACGAGATG-3′ (reverse)
- GAPDH: 5′-GACTCATGACCACAGTCCATGC-3′ (forward)
- 5′-AGAGGCAGGGATGATGTTCTG-3′ (reverse)

### CEA and CYFRA21-1 detection

The serum CEA and CYFRA21-1 levels were tested using an Elecsys-electrochemical immune assay (Roche, USA) and were detected in a cobas 8000 modular analyzer (Roche).

### Statistical analysis

All statistical analyses were carried out using the SPSS 20.0 statistical software package. Survival curves were analyzed by the Kaplan–Meier method, and a log-rank test was used to assess significance. Patients were classified into high-expression or low-expression group by using the corresponding median value as the cutoff point (fold change > 1.5). The correlation between the expression levels of RP5-977B1 and clinical parameters of patients was assayed by a Chi-square test. Receiver operating characteristic curves (ROC) was used to determine diagnostic metrics that were calculated using Delong method [20]. Student’s *t* test was used to compare between groups. In all cases, error bars represent the mean ± SD derived from three independent experiments. *P* values < 0.05 were considered statistically significant.

## Results

### RNA sequencing-based screening of dysregulated exosomal LncRNAs in NSCLC

**Fig. 1:**
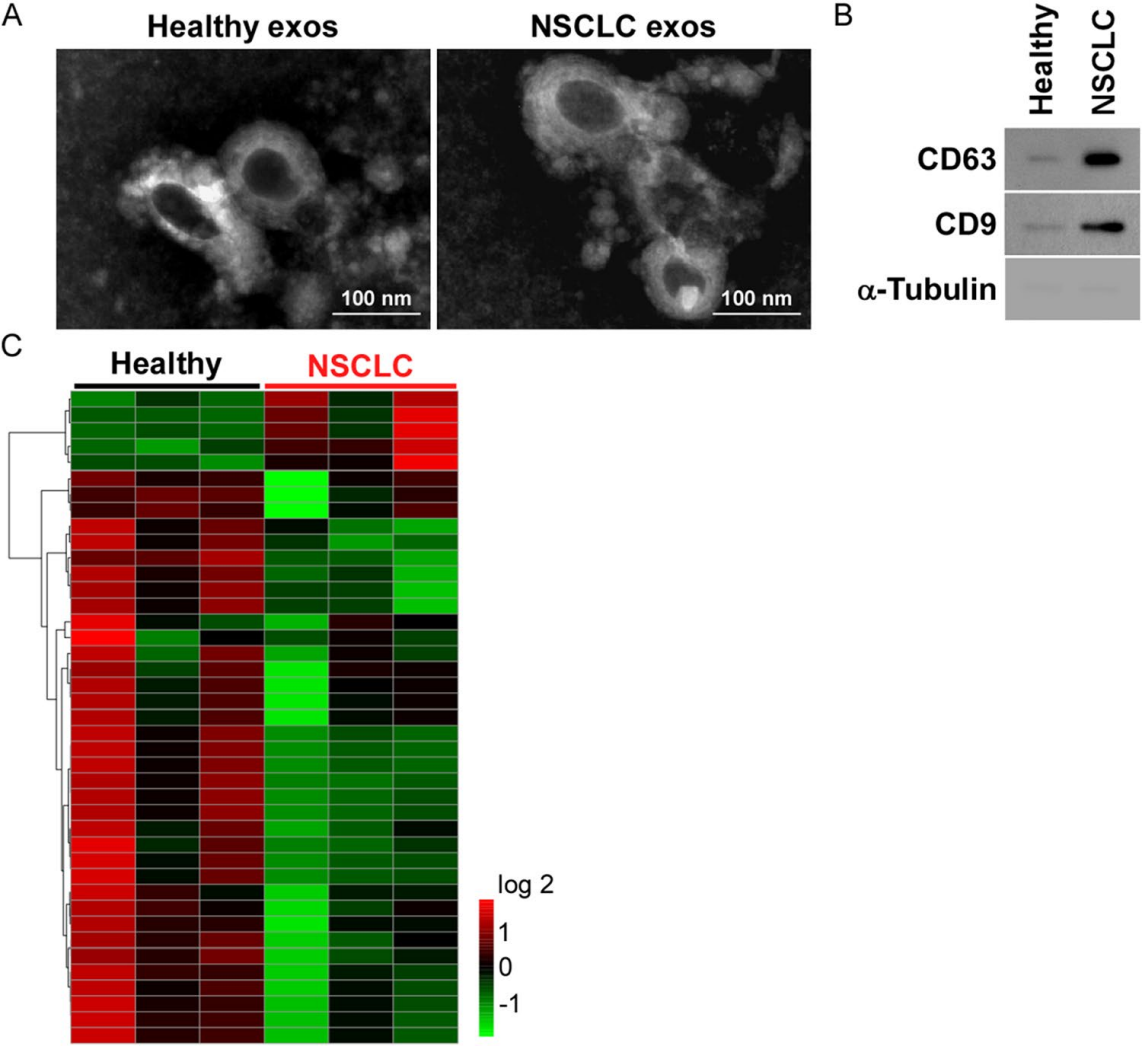
Screening and quantification of differential exosomal LncRNAs in the patients with NSCLC. **A** Representative exosomes images isolated from serum of the patients and healthy controls. Bar equals 100 nm. **B** Specific exosome marker (CD9 and CD63) and the negative control α-tubulin using western blotting assay. **C** Heatmap of the dysregulated exosomal LncRNAs in serum samples of patients with NSCLC and the healthy

To identify the dysregulated exosomal LncRNAs in NSCLC, we collected serum specimens from patients diagnosed with NSCLC (*n* = 3) and healthy controls (*n* = 3). After isolation of serum exosomes by differential ultracentrifugation, transmission electron microscopy (TEM) and NanoSight particle tracking were applied for identification and quantification of exosomes. As shown in Fig. 1A, exosomes obtained from NSCLC patients and healthy people exhibited similar typical lipid bilayer membrane morphology. Specific protein positive markers (CD9 and CD63) and negative control (α-tubulin) were used to identify the exosomes [18] (Fig. 1B). Furthermore, we found that the particle size distribution of exosomes was mainly around 30–150 nm and concentrations was enough to analyze (Supplementary Fig. 1).

Next, exosomal RNA was extracted, purified, and further analyzed via RNA sequencing (Fig. 1C and Supplementary Table 1). According to expression profiles, up-regulated exosomal LncRNAs were detected and verified at first in NSCLC.

### Verification the expression levels in the testing cohort

**Fig. 2:**
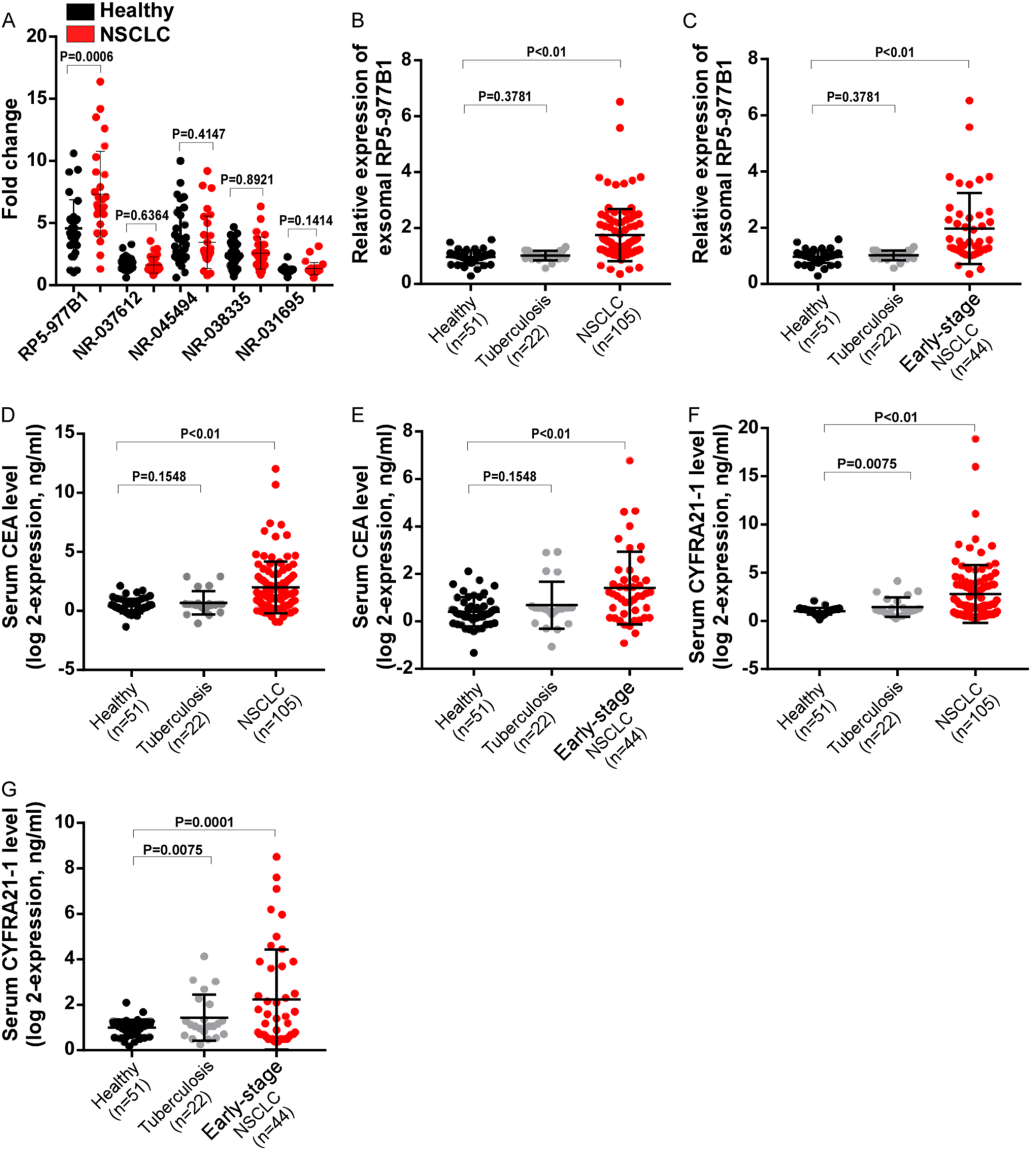
Verification the expression levels in the testing cohort. **A** Analyses of exosomal LncRNA levels in 30 cases of lung cancer patients and the healthy controls by qRT-PCR assay (unpaired *t* test). **B** Validation of exosomal RP5-977B1 in 105 cases of NSCLC patients, 22 cases of pulmonary tuberculosis patients, and 51 cases of healthy controls by qRT-PCR assay (unpaired *t* test). **C** Validation of exosomal RP5-977B1 in 44 cases of early-stage NSCLC patients, 22 cases of pulmonary tuberculosis patients, and 51 cases of healthy controls by qRT-PCR assay (unpaired *t* test). **D** Detection of serum CEA in the testing cohort by qRT-PCR assay (unpaired *t* test). **E** Detection of serum CEA in the testing cohort with early-stage NSCLC by qRT-PCR assay (unpaired *t* test). **F**, Verification of serum CYFRA21-1 in the testing cohort by qRT-PCR assay (unpaired t test). **G** Verification of serum CYFRA21-1 in the testing cohort by with early-stage NSCLC qRT-PCR assay (unpaired *t* test)

To further validate the sequencing data, we performed qRT-PCR assay to detect significantly overexpressed LncRNAs. As shown in Fig. 2A, exosomal RP5-977B1 was significantly overexpressed in NSCLC, while other assessed LncRNAs showed no significant or only weak effects, suggesting that RP5-977B1 might be the candidate molecule for further study (Fig. 2A). Pulmonary tuberculosis was the common benign pulmonary lesions that increased the difficulty of detecting NSCLC with existing detection method, so we included it in the control group. Further analysis was focused on the expression of RP5-977B1 in the testing sets with 178 serum specimens, qRT-PCR assays indicated that exosomal RP5-977B1 was significantly up-regulated in patients with NSCLC and early-stage NSCLC, when compared with healthy and pulmonary tuberculosis controls (Fig. 2B, C). We next examined the expression of conventional markers Carcinoembryonic Antigen (CEA) and Cytokeratin 19 Fragment (CYFRA21-1) in the testing sets. As shown in Fig. 2D–G, CEA and CYFRA21-1 levels were also increased in patients with NSCLC when compared with healthy controls, while significant difference was discovered between healthy and pulmonary tuberculosis controls as well (Fig. 2D–G).

### Diagnostic power of exosomal RP5-977B1 in the testing cohort

**Fig. 3:**
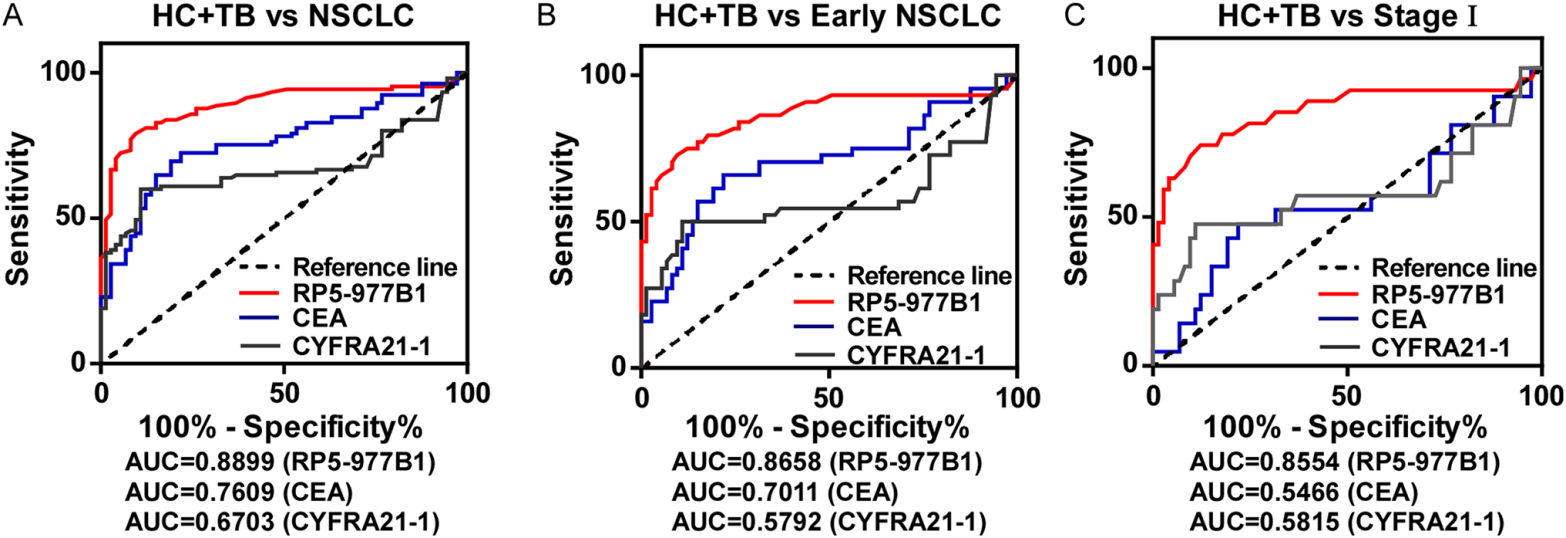
Diagnostic value of exosomal RP5-977B1 in the early-stage testing cohort. **A** ROC curve of exosomal RP5-977B1, serum CEA and CYFRA21-1 in NSCLC and controls. **B** ROC curve of exosomal RP5-977B1, serum CEA and CYFRA21-1 in the early-stage patients and controls. **C** ROC curve of exosomal RP5-977B1, serum CEA and CYFRA21-1 in patients with stage I and controls

Furthermore, the receiver operating characteristic (ROC) curve (AUC) was drawn to assess the diagnostic value of exosomal RP5-977B1. Exosomal RP5-977B1 revealed an AUC value of 0.8899 (*P* < 0.001) in distinguishing patients with NSCLC from the healthy and patients with pulmonary tuberculosis, while serum CEA and CYFRA21-1 shown an AUC = 0.7609 (*P* < 0.001) and 0.6703 (*P* = 0.0001), respectively (Fig. 3A, Table 1). The AUC value of RP5-977B1 was significantly higher than CEA and CYFRA21-1 (*P* < 0.05, Table 2). For early-stage NSCLC (stage I and II), exosomal RP5-977B1 was superior in distinguishing patients from controls (for stage I and II patients, AUC = 0.8658, *P* < 0.001), while serum CEA and CYFRA21-1 revealed an AUC = 0.7011 (*P* = 0.0003) and 0.5792 (*P* = 0.5792), respectively (Fig. 3B, Tables 1, 2). Diagnostic advantage was also achieved for stage I patients (for exosomal RP5-977B1, AUC = 0.8377, *P* < 0.001, for serum CEA, AUC = 0.5694, *P* = 0.3920, for serum CYFRA21-1, AUC = 0.5792, *P* = 0.1521), indicating that exosomal RP5-977B1 exhibited advantages in the diagnosis of early-stage NSCLC (Fig. 3C, Tables 1, 2).

**Table 1:** Diagnostic efficiency of ROC curves in the testing cohort

Comparison	AUC [95% CI]	*P* value	Sensitivity (%)	Specificity (%)
NSCLC vs TB + HC				
RP5-977B1	0.8899 [0.8398–0.94]	< 0.001	82.86	84.93
CEA	0.7609 [0.6906–0.8312]	< 0.001	72.38	78.08
CYFRA21-1	0.6703 [0.5898–0.7508]	0.0001	60.95	79.45
Stages I and II and TB + HC				
RP5-977B1	0.8658 [0.7861–0.9455]	< 0.001	81.82	75.34
CEA	0.7011 [0.6153–0.8343]	0.0003	70.45	64.38
CYFRA21-1	0.5792 [0.4549–0.7035]	0.1521	54.55	63.01
Stage I and TB + HC				
RP5-977B1	0.8377 [0.7239–0.9514]	< 0.001	79.17	73.58
CEA	0.5694 [0.3908–0.748]	0.3920	52.94	73.58
CYFRA21-1	0.5792 [0.4549–0.7035]	0.1521	50.00	89.04

*NSCLC* non-small cell lung cancer, *CI* confidence interval, *CEA* carcinoembryonic antigen, *CYFRA*21-1 cytokeratin 19 Fragment, *TB* pulmonary tuberculosis, *HC* healthy controls, *AUC* area under the curve

**Table 2:** Pairwise comparison of ROC curves in testing cohort

Comparison	95% CI	*z* statistic	*P* value
NSCLC and TB + HC			
CEA vs CYFRA21-1	− 0.0089 to 0.190	1.790	0.0735
CEA vs RP5-977B1	0.0416 to 0.216	2.892	0.0038
CYFRA21-1 vs RP5-977B1	0.121 to 0.318	4.386	< 0.0001
Stages I and II and TB + HC			
CEA vs CYFRA21-1	− 0.0816 to 0.091	0.107	0.9144
CEA vs RP5-977B1	0.0910 to 0.301	3.655	0.0003
CYFRA21-1 vs RP5-977B1	0.0903 to 0.312	3.561	0.0004
Stage I and TB + HC			
CEA vs CYFRA21-1	− 0.108 to 0.128	0.163	0.8705
CEA vs RP5-977B1	0.144 to 0.430	3.924	0.0001
CYFRA21-1 vs RP5-977B1	0.137 to 0.417	3.881	0.0001

*NSCLC* non-small cell lung cancer, *CI* confidence interval, *CEA* carcinoembryonic antigen, *CYFRA*21-1 cytokeratin 19 fragment, *TB* pulmonary tuberculosis, *HC* healthy controls

### Detection of exosomal RP5-977B1 in the validation cohort

**Fig. 4:**
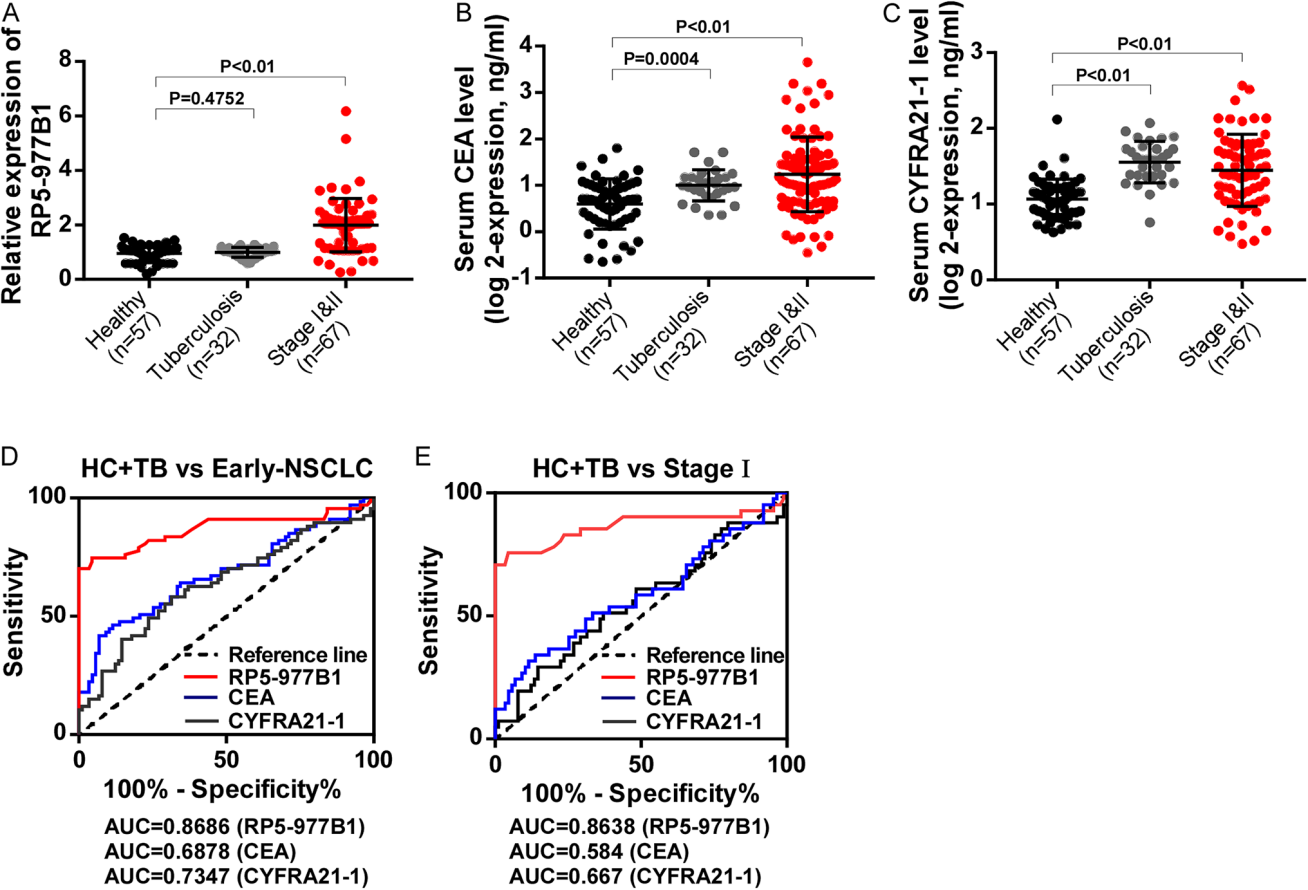
Detection of exosomal RP5-977B1 in the early-stage validation cohort. The expression of exosomal RP5-977B1 (**A**) serum CEA (**B**) and CYFRA21-1 (**C**) in healthy controls, patients with pulmonary tuberculosis and NSCLC with stage I and II in the validation cohort (unpaired *t* test). **D** ROC curve of exosomal RP5-977B1, serum CEA and CYFRA21-1 in the early-stage patients and controls. **E** ROC curve of exosomal RP5-977B1, serum CEA and CYFRA21-1 in patients with stage I and controls

We further detected the expression of exosomal RP5-977B1 in a multicentric early-stage cohort with 156 serum specimens (67 NSCLC patients with early stage and 89 healthy and tuberculosis controls). As shown in Fig. 4A–C, exosomal RP5-977B1 was highly expressed in NSCLC patients with stage I and II, while not in control patients with pulmonary tuberculosis (Fig. 4A). Serum CEA and CYFRA21-1 also showed elevated levels in early-stage NSCLC, but failed to distinguish patients with pulmonary tuberculosis from NSCLC (Fig. 4B, C). Corresponding to these results, the AUCs of RP5-977B1, CEA and CYFRA21-1 in the validation cohort were 0.8686 (*P* < 0.001), 0.6878 (*P* < 0.001) and 0.6361 (*P* = 0.0037) in distinguishing early-stage NSCLC from controls (Fig. 4D). Furthermore, the AUCs of RP5-977B1, CEA and CYFRA21-1 were 0.8638 (*P* < 0.001), 0.5840 (*P* = 0.1261) and 0.6670 (*P* = 0.3174) in distinguishing patients with stage I from controls (Fig. 4E, Table 3). The AUC value of RP5-977B1 was significantly higher than CEA and CYFRA21-1 (*P* < 0.05, Table 4), diagnostic accuracy of exosomal RP5-977B1 for early-stage NSCLC was also verified in the validation cohort.

**Table 3:** Diagnostic efficiency of ROC curves in validation cohort

Comparison	AUC [95% CI]	*P* value	Sensitivity (%)	Specificity (%)
Stage I and II and TB + HC				
RP5-977B1	0.8658 [0.7861–0.9455]	< 0.001	82.09	76.40
CEA	0.6878 [0.6104–0.7653]	< 0.001	67.39	66.67
CYFRA21-1	0.6361 [0.5456–0.7266]	0.0037	58.21	68.54
Stage I and TB + HC				
RP5-977B1	0.8638 [0.7748–0.9527]	< 0.001	80.49	77.53
CEA	0.5840 [0.4712–0.6967]	0.1261	51.22	66.67
CYFRA21-1	0.5547 [0.4435–0.6659]	0.3174	51.22	62.92

*AUC *area under the curve, *CI* confidence interval, *CEA* carcinoembryonic antigen, *CYFRA*21-1 cytokeratin 19 fragment, *TB* pulmonary tuberculosis, *HC* healthy controls

**Table 4:** Pairwise comparison of ROC curves in validation cohort

Comparison	95% CI	*z* statistic	*P* value
Stages I and II and TB + HC			
CEA vs CYFRA21-1	− 0.0816 to 0.091	0.107	0.9144
CEA vs RP5-977B1	0.0910 to 0.301	3.655	0.0003
CYFRA21-1 vs RP5-977B1	0.0903 to 0.312	3.561	0.0004
Stage I and TB + HC			
CEA vs CYFRA21-1	− 0.108 to 0.128	0.163	0.8705
CEA vs RP5-977B1	0.144 to 0.430	3.924	0.0001
CYFRA21-1 vs RP5-977B1	0.137 to 0.417	3.881	0.0001

*CI* confidence interval, *CEA* carcinoembryonic antigen, *CYFRA*21-1 cytokeratin 19 fragment, *TB* pulmonary tuberculosis, *HC* healthy controls

### Validation the existing pattern of RP5-977B1

**Fig. 5:**
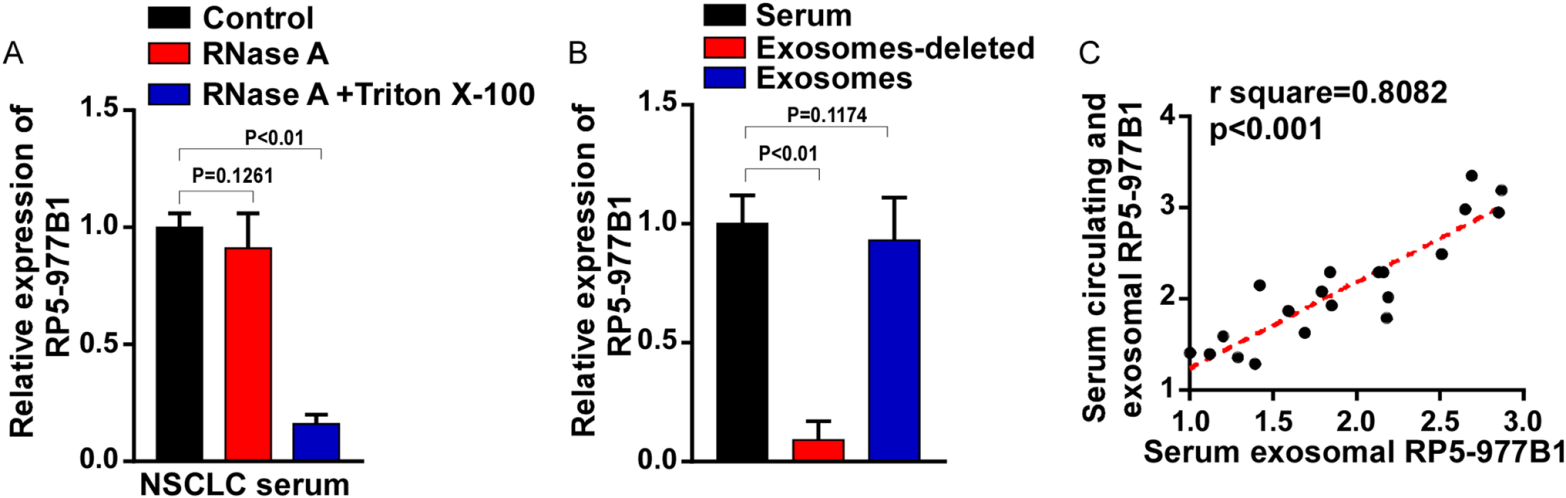
Validation the existing pattern of RP5-977B1. **A** qRT-PCR analysis of RP5-977B1in the serum of NSCLC patients treated with RNase A (2 mg/ml) or combined with Triton X-100 (0.1%) (unpaired *t* test). **B** The volume of total serum was equal to the volume of exosomes-deleted plus exosomal serum, and originated from the same patient. qRT-PCR analysis of relative expression of RP5-977B1 in the total serum, exosomes or exosomes-deleted serum (unpaired *t* test). The expression of RP5-977B1 was normalized to corresponding GAPDH. **C** Correlations between exosomal circulating RP5-977B1 and serum RP5-977B1 (Pearson’s correlation test)

We next explored whether RP5-977B1 was mainly existed in exosomes. To this end, 21 cases of NSCLC serum samples were directly incubated with RNase A, or both RNase A and Triton X-100. As shown in Fig. 5A, the levels of RP5-977B1 were constant when treated with RNase A, but decreased significantly upon RNase A and Triton X-100 treatment (Fig. 5A). It was indicated that RP5-977B1 was mainly encapsulated by exosomes rather than released directly. Moreover, the amount of RP5-977B1in serum was predominant in the exosomes, but significantly reduced in exosomes-depleted serum (Fig. 5B). As expected, the levels of RP5-977B1 in serum exosomes were positively correlated with peripheral blood according to the results of correlation coefficient (*r*^2^ = 0.8082, *P* < 0.01) (Fig. 5C). Taken together, these results suggested that exosome was the main existing pattern of RP5-977B1 in the peripheral blood.

### Exosomal RP5-977B1 levels indicated worse prognosis of NSCLC patients

The significant increase of exosomal RP5-977B1 in early-stage NSCLC prompted us to investigate whether RP5-977B1 could predict the prognosis in NSCLC. To achieve this end, we collected the clinical information of the patients, which was summarized in Table 5. By analyzing these data, we found that RP5-977B1 level was significantly correlated with tumor stage and distant metastasis, which contributed to the further intensive study (Table 6).

**Table 5:** Clinicopathologic characteristics of patient samples and expression of RP5-977B1 in lung cancer patients from CCGMU cohort

Characteristics	Number of cases	Percentage (%)
Gender		
Male	76	72.4
Female	29	27.6
Age (years)		
> 60	56	53.3
≤ 60	49	46.7
Clinical stage		
I	21	20.0
II	23	21.9
III	18	17.1
IV	43	41.0
T classification		
T1	24	22.9
T2	40	38.1
T3	13	12.4
T4	18	17.1
N classification		
N0	37	35.2
N1	12	11.4
N2	31	16.4
N3	24	22.9
Unknown	1	1.0
Distant metastasis		
Yes	43	41.0
No	62	58.1
RP5-977B1Expression		
RP5-977B1 low	52	49.5
RP5-977B1 high	53	50.5

**Table 6:** Correlation between clinicopathologic features and expressions of RP5-977B1 in lung cancer patients from CCGMU cohort

Characteristics	RP5-977B1		χ^2^	Chi-square test
	Lower expression	Higher expression		
Gender				
Male	37	39	0.078	0.781
Female	15	14		
Age (years)				
> 60	27	29	0.082	0.774
≤ 60	25	24		
Clinical stage				
I	11	10	14.930	0.002
II	11	12		
III	2	16		
IV	28	15		
T classification				
T1	11	13	1.728	0.631
T2	20	20		
T3	4	9		
T4	7	11		
N classification metastasis				
N0	20	17	2.034	0.565
N1	6	6		
N2	17	14		
N3	9	15		
Unknown	1			
Distant metastasis				
Yes	28	15	7.083	0.008
No	24	38		

**Fig. 6:**
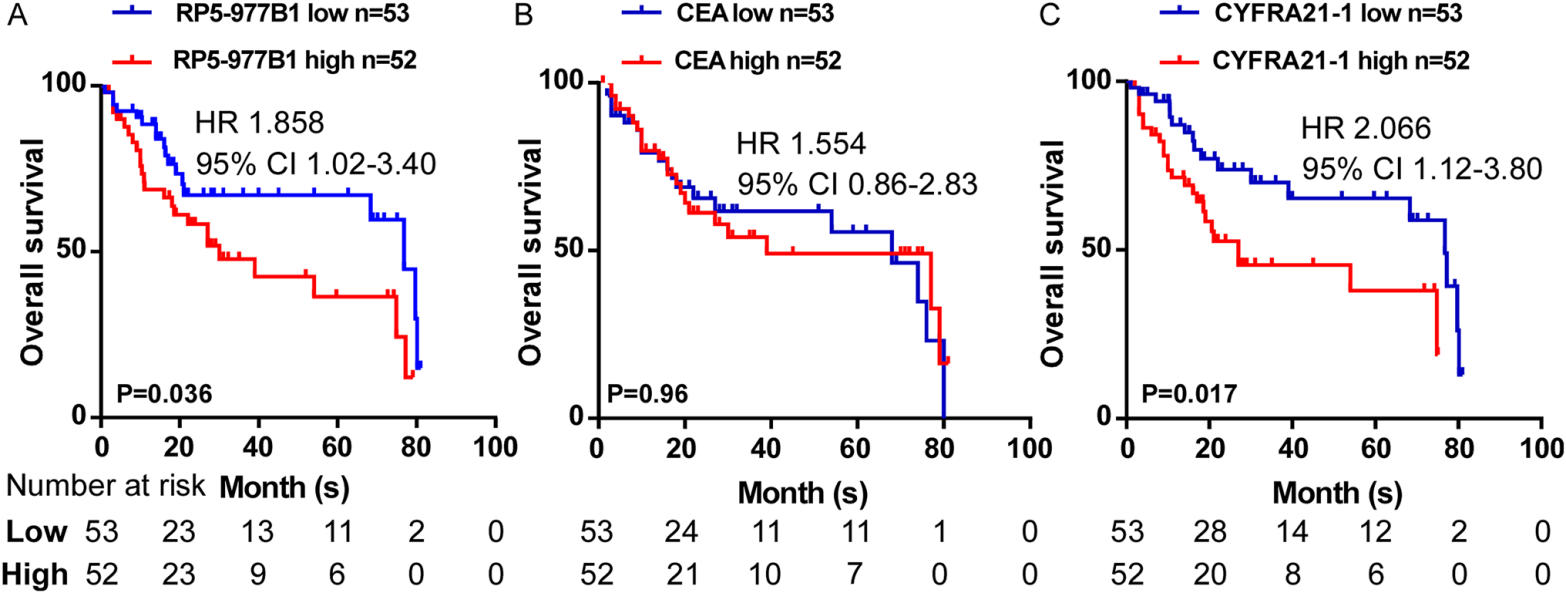
Prognostic value of exosomal RP5-977B1. Kaplan–Meier survival analysis of overall survival in 105 cases of CCGMU lung cancer cohorts based on exosomal RP5-977B1 (**A**) serum CEA (**B**) and CYFRA21-1 (**C**) expression (log-rank test)

Stratified by a median cutoff of RP5-977B1 expression, we found that higher RP5-977B1 expression indicated shorter overall survival than those lower levels of RP5-977B1 (*P* = 0.036) (Fig. 6A). Serum CEA and CYFRA21-1 were also reported to indicate the prognosis in the previous study [21, 22]. As shown in Fig. 6B, C, high CEA and CYFRA21-1 levels were related with shorter survival time (Fig. 6B, C). Collectedly, these results indicated that exosomal RP5-977B1 might have the potential to predict the prognosis of NSCLC.

## Discussion

Our current study reports a novel exosomal LncRNA, RP5-977B1, which was up-regulated in NSCLC, as evidenced by RNA sequencing and qRT-PCR assay. With encapsulating into exosomes, RP5-977B1 has high stability and easy access to monitor in circulation, which contributed to be the promising candidate molecules for dynamic detection. ROC curves data supported that RP5-977B1 could discriminate patients with early-stage NSCLC from controls with privileging sensitivity and specificity. Furthermore, survival analyses indicated that high level of RP5-977B1 was associated with shorter survival time in NSCLC. Collectedly, our study demonstrated that exosomal LncRNA RP5-977B1 showed the potential to be the biomarker for early diagnosis and prognosis prediction in NSCLC.

With unique properties of stability and dynamic detection, exosomes develop to be the promising carrier for various molecules, in particular LncRNAs [23, 24]. It was reported that exosomes transport a preponderance of LncRNAs, which almost reached 20.19% of exosomal RNAs extraction in the plasma of castration-resistant prostate cancer patients [25]. In this study, we find that LncRNA RP5-977B1 was exported by exosomes and exhibited elevated levels in NSCLC through RNA sequencing. Analyzing the sequencing results, the number of up-regulated LncRNAs was listed limited, the cause may be largely due to the insufficient sequencing depth. More overexpressed LncRNAs are expected to study and excavate further. RP5-977B1 was found to be a highly conservative LncRNA that located on chromosome 20 and contains two exons. Ensembl Genome Browser (version 90; http://www.ensembl.org/index.html) showed that the full length of RP5-977B1 is 714 nt, which was equipped with the basic characteristics of an LncRNA. Of particular note, it is the pioneering study to investigate LncRNA RP5-977B1 and explore its diagnostic and prognostic potential for NSCLC.

Operation is the most effective therapy for early-stage NSCLC with clear surgical indication [26]. Due to initial diagnosis at advanced stage, less than 30 percent of patients accept surgically resectable tumors [27, 28]. Studies analyzing the results of different treatment suggest that mortality rate would be further decreased if diagnosed at early stage. Conventional screening of NSCLC with MRI or CT would be exorbitantly expensive and relevant to high rates of false positives, while tumor markers provide complementary risk assessment for the clinic decision making. Patients with NSCLC show high levels of tumor markers CEA and Cyfra21-1 [29–31], while they are not specifically overexpressed at early stage, which facilitates the establishment of new and effective diagnostic or prognostic biomarkers. Exosomal RP5-977B1 was overexpressed in serum of early-stage NSCLC with reliable sensitivity and specificity in distinguishing patients of NSCLC from the healthy and pulmonary tuberculosis, exhibiting satisfactory potential for diagnostic markers.

The heterogeneity of NSCLC results in that even if patients at same stage and accept the same therapy, the prognosis can be the opposite [32, 33]. Accumulating evidence has indicated that specific LncRNA expression was correlated with clinical features in various types of cancers, supporting the utility of LncRNA in prognosis of the disease [34, 35]. Moreover, detection of peripheral blood exosomal LncRNA makes repeated measurements minimally invasive and reveals the survival status for patients over time. In this study, we found that high levels of exosomal RP5-977B1 indicated worse prognosis and was significantly correlated with tumor stage and distant metastasis. Cedrés et al. has reported that CEA and Cyfra21-1 are a prognostic indicator of poor survival in NSCLC [21, 22, 36]. Our study showed that CEA and Cyfra21-1 were also positively related with worse prognosis, while CEA indicated no statistical difference, may be due to the limited sample size. Conclusively, exosomal RP5-977B1 may be the promising candidate serum-based biomarkers for dynamical monitoring the prognosis in NSCLC.

Given the clinical significance of RP5-977B1in NSCLC, we find that exosomal RP5-977B1 can be novel diagnostic and prognostic biomarkers for NSCLC patients. Exosomes are the leading carrier for LncRNA RP5-977B1 cargo, with better stability and reproducible detection, exosomal RP5-977B1 is expected to become non-invasive biomarkers for NSCLC.

## Conclusions

To date, biomarker for early diagnosis was not fully explored in NSCLC. With stabilization and considerable tumor specificity, exosomal LncRNA developed to be the ideal tumor diagnostic marker. Our findings suggest exosomal LncRNA RP5-977B1 had favorable sensitivity and specificity for early diagnosis in NSCLC, which supposed to be the novel diagnostic biomarker in the future. Exosomal RP5-977B1 level was also negatively related with prognosis of NSCLC, and correlated with tumor stage and distant metastasis, indicating its potential for serum-based biomarker of prognosis. Our results provide the new sights into early diagnosis and drug therapy targets in NSCLC.

## Supplementary Information

Below is the link to the electronic supplementary material.

Supplementary file1 (PDF 32 KB)

Supplementary file2 (XLSX 15 KB)
