# Supplementary material for: Exosomal LncRNA RP5-977B1 as a novel minimally invasive biomarker for diagnosis and prognosis in non-small cell lung cancer
Source: Int J Clin Oncol. 2022 Apr 28;27(6):1013–24. doi: 10.1007/s10147-022-02129-5 (PMC9120093; doi:10.1007/s10147-022-02129-5)
Supplement: Supplementary file 1 — Supplementary file1 (PDF 32 KB) [file 10147_2022_2129_MOESM1_ESM.pdf]

Supplementary Figure 1

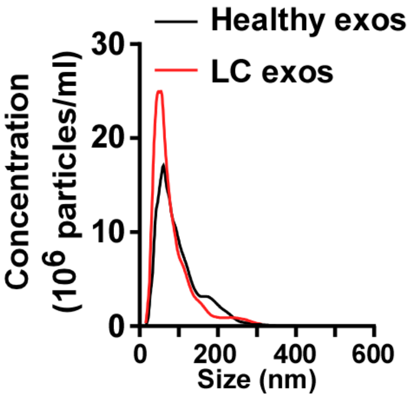

Supplementary Figure 1. NanoSight particle tracking analysis of the size distributions and number of exosomes.
